# Desensitizing nicotinic agents normalize tinnitus-related inhibitory dysfunction in the auditory cortex and ameliorate behavioral evidence of tinnitus

**DOI:** 10.3389/fnins.2023.1197909

**Published:** 2023-05-25

**Authors:** Madan Ghimire, Rui Cai, Lynne Ling, Kevin A. Brownell, Kurt W. Wisner, Brandon C. Cox, Troy A. Hackett, Thomas J. Brozoski, Donald M. Caspary

**Affiliations:** ^1^Department of Pharmacology, Southern Illinois University School of Medicine, Springfield, IL, United States; ^2^Department of Otolaryngology, Head and Neck Surgery, Southern Illinois University School of Medicine, Springfield, IL, United States; ^3^Department of Hearing and Speech Sciences, Vanderbilt University Medical Center, Nashville, TN, United States

**Keywords:** tinnitus, nicotinic cholinergic receptor, primary auditory cortex, pyramidal neuron, VIP neuron, sazetidine-A, varenicline

## Abstract

Tinnitus impacts between 10–20% of the population. Individuals most troubled by their tinnitus have their attention bound to and are distracted by, their tinnitus percept. While numerous treatments to ameliorate tinnitus have been tried, no therapeutic approach has been clinically accepted. The present study used an established condition-suppression noise-exposure rat model of tinnitus to: (1) examine tinnitus-related changes in nAChR function of layer 5 pyramidal (PNs) and of vasoactive intestinal peptide (VIP) neurons in primary auditory cortex (A1) and (2) examine how the partial desensitizing nAChR agonists, sazetidine-A and varenicline, can act as potential therapeutic agents in the treatment of tinnitus. We posited that tinnitus-related changes in layer 5 nAChR responses may underpin the decline in attentional resources previously observed in this animal model (Brozoski et al., 2019). *In vitro* whole-cell patch-clamp studies previously revealed a significant tinnitus-related loss in nAChR-evoked excitatory postsynaptic currents from A1 layer 5 PNs. In contrast, VIP neurons from animals with behavioral evidence of tinnitus showed significantly increased nAChR-evoked excitability. Here we hypothesize that sazetidine-A and varenicline have therapeutic benefits for subjects who cannot divert their attention away from the phantom sound in their heads. We found that sazetidine-A or varenicline normalized tinnitus-related reductions in GABAergic input currents onto A1 layer 5 PNs. We then tested sazetidine-A and varenicline for the management of tinnitus using our tinnitus animal model. Subcutaneous injection of sazetidine-A or varenicline, 1 h prior to tinnitus testing, significantly decreased the rat’s behavioral evidence of tinnitus in a dose-dependent manner. Collectively, these results support the need for additional clinical investigations of partial desensitizing nAChR agonists sazetidine-A and varenicline for the treatment of tinnitus.

## Introduction

Tinnitus is a phantom ringing sound experienced in the absence of an acoustic stimulus. Subjects most disturbed by their tinnitus also show bimodal abnormalities of selective attention. These individuals are bound to their tinnitus percept while also being distracted by their tinnitus, resulting in impaired attentional function (Burton et al., 2012; Roberts et al., 2013; McKenna et al., 2014). As a result, tinnitus is believed to impact many aspects of selective attention (Hallam et al., 2004; Rossiter et al., 2006; McKenna et al., 2014). Tinnitus sufferers show poor performance on cognitive tasks that demand selective attention compared to individuals without tinnitus (Hallam et al., 2004; Rossiter et al., 2006; Stevens et al., 2007). Brozoski et al. (2019) found significant behavioral deficits in selective auditory attention in the rodent model of chronic tinnitus used in the present study.

When attention is required, cholinergic neurons in the basal forebrain (BF) are activated, to release acetylcholine (ACh) onto neurons in the auditory cortex (A1) (Metherate, 2011; Schofield and Hurley, 2018). ACh then activates pre- and postsynaptic receptors in a subpopulation of A1 neurons (Metherate and Ashe, 1993; Nelson and Mooney, 2016; Ghimire et al., 2020). ACh has been found to increase the activity of certain A1 neurons via homomeric (α7), heteromeric (primarily α4β2) neuronal nicotinic acetylcholine receptors (nAChRs), and M1 type muscarinic receptors (Hilscher et al., 2017; Colangelo et al., 2019; Fu et al., 2019). A1 contains complex microcircuitry consisting of at least two types of excitatory pyramidal neurons (PNs) and at least three different types of inhibitory neurons, parvalbumin-positive (PV), vasointestinal peptide-positive (VIP) and somatostatin-positive (SOM), which can modulate sensory input and output through direct/indirect effects on excitatory neurons (Xu et al., 2013; Blackwell and Geffen, 2017; Hattori et al., 2017; Phillips et al., 2017). Previous studies indicated that inhibitory VIP neurons can be activated by the release of ACh, which in turn inhibit PV and SOM neurons favoring the excitability of PNs via disinhibitory mechanisms (Phillips et al., 2017; Askew et al., 2019). Therefore, ACh signaling should be considered an important modulator of excitatory neuronal function in the normal, as well as in pathological auditory pathways (Roberts et al., 2013). For example, tinnitus-related increases in excitability at multiple subcortical and cortical structures may reflect pathological changes in nAChR function (Norena, 2011; Auerbach et al., 2014; Ghimire et al., 2020; Fuksa et al., 2022).

Identification of cholinergic agents that can modulate tinnitus-related attentional dysfuntion by reducing tinnitus-related hyperexcitability is a goal of the present study. To date, multiple tinnitus interventions, including use of anticonvulsants, antidepressants, glutamatergic and GABAergic agents have been examined without consistent success (Dobie, 2003, 2004; Langguth et al., 2011, 2019). Human psychoacoustic studies find that individuals experiencing chronic and persistent tinnitus report dramatic decreases in their tinnitus percept when their focus was shifted away from tinnitus (Roberts et al., 2013). While behavioral interventions such as sound therapy utilize the principles and strategies of attentional shift, drug studies have not attempted tinnitus management by diverting attention from the phantom percept (Argstatter et al., 2012). The present studies sought to characterize tinnitus-related changes in nAChR function in A1 layer 5 PNs and VIP neurons. Sazetidine-A and varenicline are partial nAChR desensitizing agonists that have been reported to improve altered attentional and cognitive functions in animal studies. In animal pain models, sazetidine-A and varenicline were found to be effective in the management of chronic neuropathic pain, which may share pathological features with tinnitus (Cucchiaro et al., 2008; Møller, 2011; AlSharari et al., 2012).

## Methods

Animals: 58 wild-type (WT) and 17 *VIP^Cre^:Rosa26^tdTomato^* adult Long Evans rats of 3–4 months of age were entered into the studies and used in a series of subcellular and behavioral experiments at 7–9 months of age. Homozygote *VIP^Cre^* males (*HsdSage:LE-VIP^em1(T2A-Cre)Sage^*) were bred in house with homozygote *Rosa26^loxP-stop-loxP-tdTomato^* reporter females (*HsdSage:LE-Rosa26^em1(tdTomato)Sage^*). Both lines were purchased from Envigo (Indianapolis, IN). Rat toes were tattooed to identify the animal and genotyping was performed by Transnetyx, Inc. (Memphis, TN). Experimental details of animal usage are shown in Table 1. To allow for individual dietary restriction, animals were single-housed and diet-restricted from the time of noise-exposure until tinnitus assessment was complete. A reversed light/dark cycle was maintained in order to conduct behavioral studies during the animals’ active periods. All experiments were performed according to Southern Illinois University School of Medicine Institutional Animal Care and Use Committee (IACUC) approved protocol (41-021-007).

**Table 1:** Animals used in the study.

Animals	Wild-type		VIP^Cre^:Rosa26^tdTomato^	
	Sham-exposed	Noise-exposed	Sham-exposed	Noise-exposed
ABR threshold shift at 16 kHz (Mean ± SD dB)	3.49 ± 8.33	17.3 ± 14.8	2.1 ± 4.4	21.76 ± 8.1
Electrophysiological studies (Rats/Neurons)	14/42	13/43	10/36	5/14
Drug studies	13	13		
Anatomical studies	5		2	
Total animals used	32	26	12	5

## Behavioral model of tinnitus

### Hearing assessment and noise exposure

**Figure 1:**
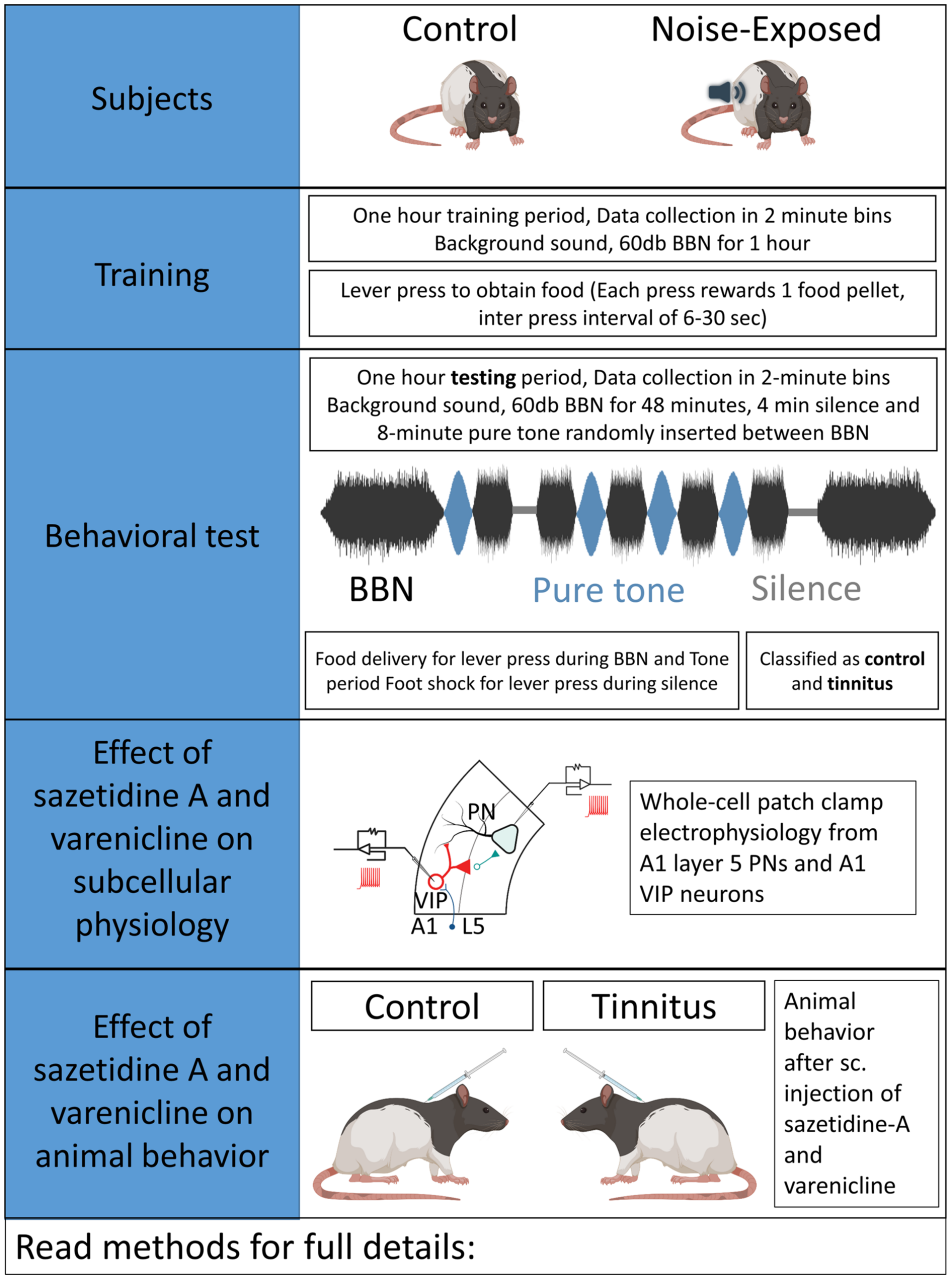
Experimental design.

Auditory brainstem response (ABR) thresholds were measured to ensure normal hearing in each animal used in the present studies. Three to 4-month-old rats were anaesthetized using a cocktail of ketamine (90 mg/kg)/xylazine (7 mg/kg) and pre- and post noise-exposure ABRs were obtained for pure tones at 8, 10, 12, 16, 20, 24, and 32 kHz, presented in 10 dB increments between 10 and 80 dB (SPL re 20 μPa). Acoustic systems were calibrated off-line using either a 1/4 or 1/5 inch Brüel & Kjær microphone (Naerum, Denmark). Methods used in the present study were identical to those used in prior studies by Bauer and Brozoski (Bauer et al., 1999; Bauer and Brozoski, 2001; Brozoski et al., 2013). Control/unexposed and noise-exposed animals were similarly tested (Figure 1). Half of the rats were unilaterally noise-exposed as detailed below, with control rats left unexposed and identically treated. Briefly, rats were unilaterally exposed to narrow band noise for 1 h, with a peak level of 116 dB (SPL), centered at 17 kHz, falling to ambient levels at 8 kHz and 24 kHz, with the contralateral ear canal blocked (Bauer and Brozoski, 2001).

### Tinnitus assessment

**Figure 2:**
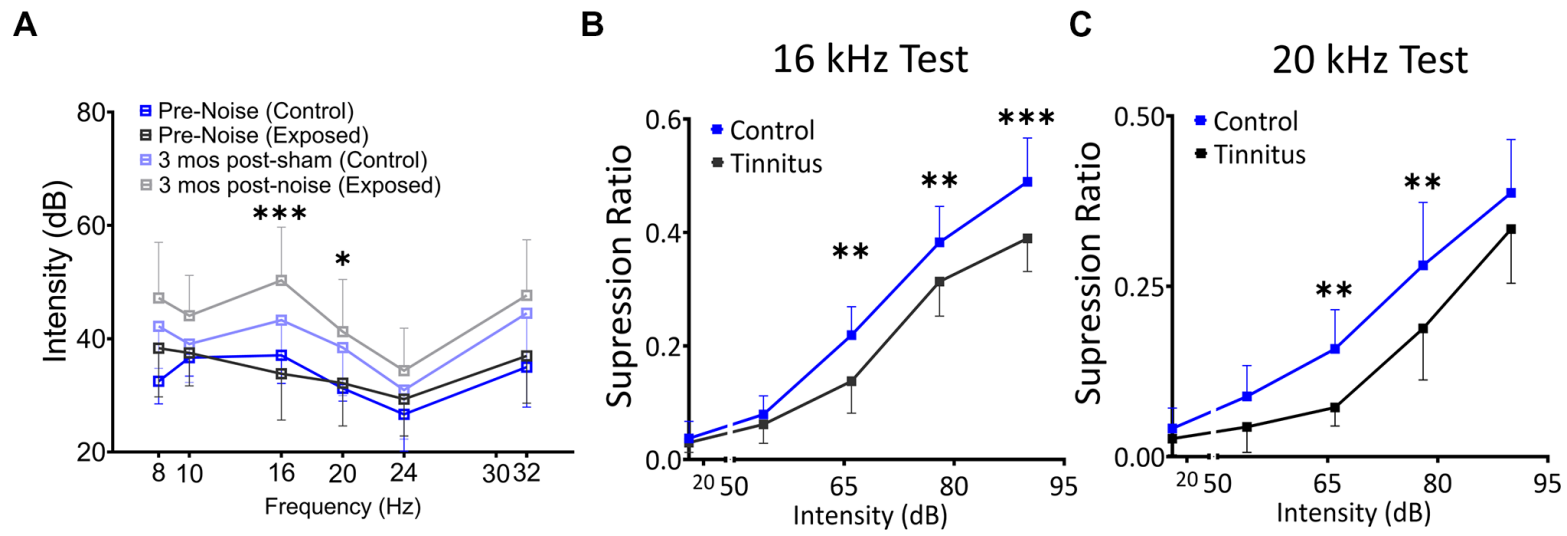
Noise exposure model of tinnitus shows threshold shifts adjacent to the exposure frequency and shifts in suppression ratios, suggestive of a decreased ability to identify silence. **(A)** ABR thresholds before and 3–4 month after 1 h of 116 dB 17 kHz centered narrowband unilateral noise exposure. The noise-exposed ear shows increased ABR thresholds around the exposure frequency (two black and grey lines). The unexposed ear shows no significant differences between groups (two blue lines). **(B,C)** Suppression ratios were calculated as a function of tone (16 kHz in B or 20 kHz in C) discrimination ability from silence (*n* = 22 control, 13 exposed tinnitus). A clear separation between control and exposed tinnitus rats were observed. ^*^ *p* < 0.05, ^**^ < 0.01, ^***^ < 0.001 Two-way ANOVA with a Bonferroni post-hoc test.

An operant conditioned-suppression procedure determined the animal’s perception of test tones and silent periods embedded in ambient, low level (60 dB, SPL) broad-band noise (BBN). Using modified operant test chambers, diet-restricted animals were trained daily to lever press to acquire food pellets and were required to discriminate between the presence and absence of sound. During the training sessions animals body weight and food acquired were monitored daily. If the body weight fell below 80% of the animals original body weight, 3–5 grams of food was supplemented at the end of the behavioral sessions. Once the animals were fully trained, they were acclimated to an hour-long tinnitus testing session composed of four 2-min-long 16 or 20 kHz pure- tones of varying intensity and two 2-min-long silence periods randomly inserted in a 48-min-long BBN noise at 60 dB. Testing sessions required animals to discriminate between BBN noise, pure-tone or silence period. Food rewards were provided for every lever press meeting the criteria during BBN noise and pure-tone period while a brief foot-shock was provided for lever presses during silence period. Foot shock provided negative reinforcement to the animals and quickly shaped their auditory discrimination. Animals able to identify silence suppressed lever pressing during the silence period. However, animals unable to identify silence would suppress lever pressing during both silence and tone periods. An operant suppression ratio was calculated using the formula: R = A/(A + B) where R = Suppression ratio; A = lever press during 16/20 kHz tone period, and B = lever press following (2 min bin) tone period. Three criteria had to be met for individual-subject data to be included for further analysis: (1) There had to be a minimum of 200 lever presses in each session, (2) mean *R* for background noise periods (i.e., baseline performance) had to be >0.4, and (3) a standard deviation of <0.2. Data from a minimum of five test sessions, for each stimulus, were averaged to derive individual and group discrimination functions for each stimulus: BBN, 16, and 20 kHz tones. Control animals showed higher suppression ratios while animals were said to show evidence of tinnitus if they had lower suppression ratios demonstrating their impaired ability to discriminate tones from silence (Figures 2B,C). Noise-exposed animals were classified as noise-exposed with behavioral evidence of tinnitus (ET) if the average suppression ratio was lower than 0.26 and animals with average suppression ratio higher than 0.26 were classified as noise-exposed without behavioral evidence of tinnitus (ENT) at 16 kHz and/or 20 kHz discrimination task. No behavioral differences were observed between wild type LE and *VIP^Cre^* rats. Once psychophysical tests were completed, an exit ABR was collected prior to *in vitro* studies.

### Drug preparation and treatment

Sazetidine-A dihydrochloride (Cat. No. 2736, batch 2A/187725) and varenicline tartrate (Cat. No. 3754, batch 3A/213769) were obtained in solid form from Tocris Bioscience (Minneapolis, MN, United States). The drugs were dissolved in sterile normal saline at a concentration of 2 mg/mL or less, and administered subcutaneously at the described doses 1 h prior to behavioral testing. Both unexposed controls and exposed tinnitus rats were treated and tested in parallel. Maximum subcutaneous treatment volumes did not exceed 0.44 ml. Dose levels of 0, 0.1, 0.5, and 1 mg/kg were tested for therapeutic effect, over a minimum of 3 consecutive days for each dose. Washout periods of 1 to 4 weeks (5 test sessions per week) intervened between drug tests, as required to recover pre-drug baseline tinnitus performance.

### Micro-injection surgery

Tinnitus tested *VIP^Cre^:Rosa26^tdTomato^* rats (8–9 months old) were anaesthetized with a cocktail of ketamine (90 mg/kg)/xylazine (7 mg/kg) (induction anesthesia) followed by 0.5–1% isoflurane (maintenance). Animals were head fixed on stereotaxic apparatus (Harvard Apparatus) and a midline incision was made to expose the skull. A craniotomy of 1.5 mm was drilled at AP −2.7 and ML −4.0 respective to Bregma corresponding to BF injection site. A 2 μL Hamilton micro-syringe containing AAV-CaMKIIa-hChR2(H134R)-EYFP (UNC Vector Core, UNC, Chapel Hill, NC) was fixed at 90-degree angle in a sagittal plane. The syringe was advanced 7.2 mm from the surface of the brain to reach substantia innominata of basal forebrain (BF), and approximately 150–200 nL of the virus was injected. Animals were allowed to recover and express the virus for a minimum of 3 weeks before being used for *in vitro* optogenetic studies.

### Cortical slice electrophysiology

LE rats (7–9 months old) were anesthetized with 4% isoflurane and cardiac perfusion was performed using ice-cold sucrose aCSF (in mM as follows: 2.5 KCl, 5 MgCl_2_, 1.23 NaH_2_PO_4_, 0.5 CaCl_2_, 250 sucrose, 26 NaHCO_3_, and 10 glucose, pH 7.4) saturated with carbogen (95% O_2_/5% CO_2_) before decapitation. After perfusion and decapitation, brains were rapidly isolated and submerged in ice cold (1°C – 2°C) sucrose aCSF, pH ~7.4, and oxygen saturation was maintained by bubbling with carbogen. aCSF composition was as follows (in mM): 125 NaCl, 3 KCl, 1 MgCl_2_, 1.23 NaH_2_PO_4_, 2 CaCl_2_, 26 NaHCO_3_, and 10 glucose. Coronal slices of 250–300 μm through A1 were sectioned using a vibratome (Pelco), following previously described protocols (Richardson et al., 2013; Ghimire et al., 2020), and incubated for 15 min at 31°C. Slices were allowed to equilibrate at room temperature (20°C – 22°C) for 60 min in carbogen-bubbled aCSF before recordings. Slices were transferred to an immersion recording chamber (2 mL), perfused at 2–3 mL/min with aCSF bubbled with carbogen, and all the recordings were performed at room temperature. Layer 5 PNs were identified morphologically using QImaging Rolera bolted to a differential interference contrast microscope (BX50WI, Olympus Optical) with a 40X water-immersion objective. Likewise, tdTomato-labeled VIP neurons were identified using a fluorescence illumination system (Lambda 421, Sutter Instruments), excitation 560 nm/emission 581 nm, attached to the DIC microscope using fluorescent optics.

### *In vitro* patch-clamp recordings

Once cells were identified, whole-cell patch-clamp recordings were performed using 3–6 mΩ fire-polished microfilament micropipettes, pulled from borosilicate glass (0.86 mm ID, 1.5 mm OD; Sutter Instruments). Two different composition of the internal solution were used: (1) (in mM): 140 potassium gluconate, 1 NaCl, 2 MgCl_2_, 5 KCl, 10 HEPES, 2 Mg-ATP, 0.3 Na-GTP, 6.88 KOH, Osm: ~300 mOsms, pH 7.3 (adjusted with Tris-Base) with calculated chloride reversal potential near −65 mV and (2) (in mM) 140 CsCl, 2 MgCl2, 4 Mg-ATP, 0.3 Na-GTP, 10 Na-HEPES, 5 QX 314 and 0.1 EGTA, with a pH of 7.25 adjusted with HCl (osmolarity, ∼290 mOsm), resulting in a Cl^−^ equilibrium potential (ECl^−^) value near 0 mV. Pipettes were attached to a Multi-clamp 700B Amplifier (Molecular Devices), and cells recorded in current-clamp mode at I = 0 or voltage-clamp mode at −65 mV at 10 kHz sampling rate. The patch pipette was attached to layer 5 PNs or layer 2/3 VIP neurons with Giga-ohm (> 4 GΩ) seal and the membrane was ruptured with a brief sharp suction. Whole-cell recordings were collected with access resistances ranging from 10–25 mΩ. Whole-cell capacitance, input resistance, and access resistance were determined by injection of a 5 mV square pulse, at 20 Hz. Exclusion criteria included the following: (1) a resting membrane potential more depolarized than −55 mV, (2) access resistance >25 mΩ, or (3) a resting input resistance <100 mΩ (for PNs only). TTL pulses, voltage commands, acquisition and display of the recorded signals were achieved with Digidata 1440A (Molecular Devices) using the Clampex 10.7 program.

### Optogenetic studies

Optogenetic studies used *VIP^Cre^:Rosa26^tdTomato^* rats that underwent viral injection at BF. 50 ms pulses of blue light (430 nm) were used to activate ChR2 with a with a ThorLabs fiber coupled LED (M430F1) with an attached 400 μm diameter fiber tip. The power of the illumation was determined to be 1.42 mW/mm^2^ at the tip of the fiber. The tip of the fiber was positioned approximately 1–2 mm above the brain slice with illumination directed at the area of interest.

### Cellular phenotyping and quantification

Multiplexed fluorescence *in situ* hybridization (FISH) was used to identify neuronal cell types in fresh frozen tissue sections (14 μm) across A1 layers. Cells were identified by detection of cell-type-specific riboprobe markers of excitatory (VGluT1, *Slc17a7*) and inhibitory (VGAT, *Slc32a1*) neuronal classes, as well as the inhibitory subtype (vasoactive intestinal peptide, *VIP*) in the same tissue sections, counterstained with DAPI. Assays were conducted in sections collected from A1 of 5 animals in each experimental group using custom RNAscope riboprobes and detection kits manufactured by Advanced Cell Diagnostics (Newark, CA), as described in prior studies from our lab (Ghimire et al., 2020). Sections were imaged at 20x using a Nikon 90i epifluorescence microscope, permitting selective detection of transcripts for each cell marker by color channel. Images were imported in HALO pathology software (Indica labs, Albuquerque, NM) for analysis. Cells expressing the transcripts of each cell marker were separately tallied across the layers of A1.

### Statistical and data analysis

Signals obtained from electrophysiological recordings were filtered at 5 kHz lowpass Gaussian filter and sEPSCs were defined as spontaneous glutamatergic currents greater than 8 pA and sIPSCs were defined as spontaneous chloride currents greater than 10 pA. Data were reduced using Clampfit 10.7 (Molecular Devices), followed by further sorting using a custom MATLAB program. ACh evoked EPSCs in L5 PNs of control and tinnitus animals were filtered at 2 kHz lowpass Gausian filter, and the peak amplitudes were measured. Since no dose–response relationship was established in exposed tinnitus animals, effect size was estimated by comparing z scores (z-score = [actual value – mean value]/standard deviation) across ACh doses, and animal groups. Statistical analysis was performed using GraphPad Prism 9, with corresponding statistical tests matched to the data noted in the figure legends. Student’s *t*-tests were used for individual sample comparison and a one-way or two-way ANOVAs with Bonferroni *post hoc* corrections were used for multiple comparisons. Data were tested for the homogeneity of variance, and *p* values corrected based on statistical significance. All comparisons with *p* value <0.05 were considered significant.

## Results

The schematic overview of these studies is illustrated in Figure 1. In an established noise exposure-induced behavioral model of tinnitus (Bauer et al., 1999), 3–4 month old wild-type Long Evans (LE) or *VIP^Cre^* rats were either sham/unexposed (controls, *n* = 46) or unilaterally (right ear) noise-exposed (*n* = 65) while protecting the left, unexposed ear. Following exposure, all animals were condition suppression trained for 3–4 months. At 6–9 months of age, rats were tested for tinnitus before being used for *in vitro* studies (LE, *n* = 42; VIP^cre^, *n* = 17) or behavioral drug studies (LE, *n* = 26). Hearing thresholds were measured before and 3 months after noise exposure with a modest shift in auditory brainstem response (ABR) thresholds only in the noise-exposed ears at frequencies adjacent to the 17 kHz exposure frequency (Figure 2A, *F* (1,172) = 8.39, *p* < 0.001, Two-way ANOVA followed by a Bonferroni post-hoc test, 16 kHz, *p* < 0.0001, 20 kHz, *p* = 0.015). Three to four months after noise exposure, animals were assessed for tinnitus using the operant suppression ratio to reflect their ability to distinguish silence from pure tones at 16 kHz and 20 kHz (exposure frequency region). All control and noise-exposed rats were given a tinnitus score based on their ability to distinguish silence from tones approximating their putative tinnitus (e.g., tones close to the exposure frequency). Based on the rat’s performance in the discrimination task, noise-exposed animals were classified as exposed tinnitus or exposed non-tinnitus (ENT) (Figures 2B,C). Rats with average suppression ratios lower than 0.26 were classified as tinnitus. Approximately 50–60% (34/65) of the exposed animals showed a decreased ability to discriminate silence from tones, with a significant separation in suppression ratios from control animals at 16 kHz (*F* (1,95) = 29.34, *p* < 0.0001, Two-way mixed ANOVA, followed by a Bonferroni post-hoc test) and 20 kHz tone (Figures 2B,C) (*F* (1, 95) = 23.85, *p* < 0.0001, Two-way mixed ANOVA, followed by a Bonferroni post-hoc test). These results were interpreted as reflecting tinnitus because the tinnitus percept would be present during silent periods and generalize to tone periods. Exposed animals that showed control-like psychophysical behavior, with near control ability to distinguish silence from tone, were categorized as ENT (Figsures 2B,C) (*F* (1, 75) = 2.6, *p* = 0.11, Two-way ANOVA, followed by a Bonferroni post-hoc test). Ghimere et al. (2023), provided the physiology/pathology basis for the present study, included recordings from ENT rats (Ghimire et al., 2023). ENT animals were not included in the present study since for the tinnitus-related functional changes tested in the present study, ENT A1 neurons’ PN group data from Ghimire et al. (2023) found no statistical differences in physiological characteristics between ENT and control animals. A total of 24 Control and 18 tinnitus animals were used for *in vitro* studies as detailed in Table 1. Whole-cell patch-clamp recordings from acute A1 slices were obtained from 85 PNs and 50 VIP neurons (Table 1).

### Layer 5 PNs show tinnitus-related decreases in nAChR-evoked postsynaptic currents

**Figure 3:**
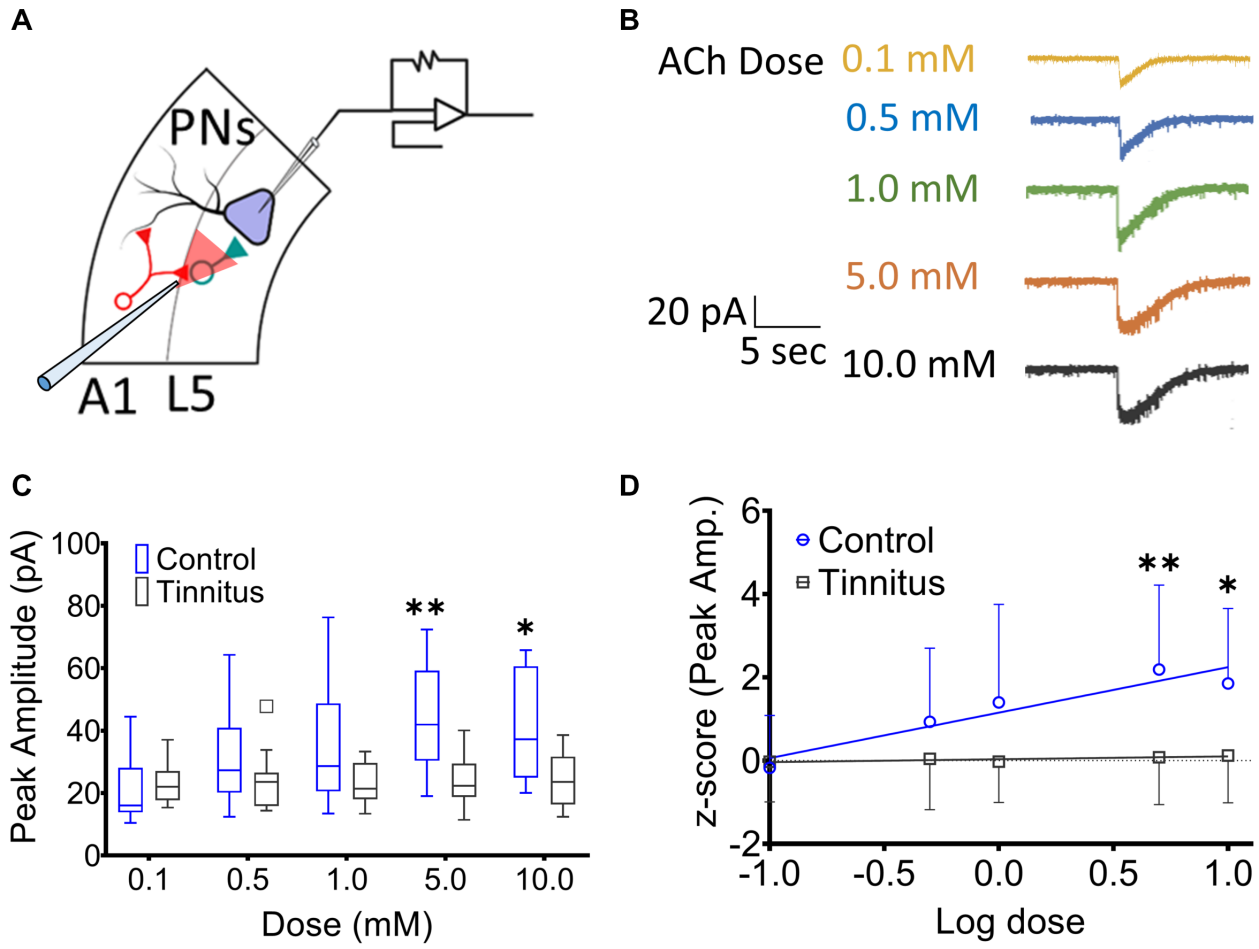
Layer 5 PN neurons in animals with tinnitus show a loss of cholinergic sensitivity. **(A)** Illustration representing whole-cell patch-clamp studies from A1 layer 5 PNs while recording responses to different doses of puffed ACh. **(B)** Exemplar traces showing responses to increasing doses of puffed ACh. **(C)** Peak amplitude of ACh evoked, nAChR-mediated, depolarizing currents were reduced in PNs from tinnitus compared to control PNs. Significant difference in evoked responses was observed with puffed ACh at 5 mM (*p* = 0.0021, Two-way ANOVA with a Bonferroni post-hoc test, *n* = 10 control, 11 tinnitus) and 10 mM (*p* = 0.03, Two-way ANOVA with a Bonferroni post-hoc test). **(D)** Unlike control (control, *r*^2^ = 0.16, *p* = 0.0028, tinnitus, *r*^2^ = 0.002, *p =* 0.71), tinnitus animals failed to show increasing nAChR responses to increasing doses of ACh. ^*^ *p* < 0.05, ^**^ < 0.01.

A1 layer 5 PNs have been shown to express α4β2 nAChR heteromeric and α7 nAChR homomeric receptors (Ghimire et al., 2020). Tinnitus-related changes in postsynaptic nAChR sensitivity were assessed by puffing increasing doses of ACh during whole-cell patch-clamp recordings from A1 layer 5 PNs in the presence of bath atropine (potassium gluconate internal solution) (ECl ~ −65 mV). Once whole-cell mode was achieved, PNs were voltage-clamped at −65 mV and a micropipette filled with 0.1–10 mM ACh was positioned approximately 30 μm away from the cell being studied (Figure 3A). ACh was puffed using picospritzer at 7.5 psi. The pipette containing one concentration of ACh was carefully withdrawn and another pipette with a higher concentration of ACh was positioned in the exact same location. nAChR-evoked currents were recorded from voltage clamped layer 5 PNs (Figure 3B). While PNs from control animals showed a linear dose–response relationship to increasing doses of ACh (*r*^2^ = 0.16, *p =* 0.0028), PNs recorded from animals with tinnitus failed to show any dose–response relationship (Figure 3C) (*r*^2^ = 0.002, *p =* 0.71). When ACh log dose was plotted against an animal’s normalized tinnitus score, the relationship between dose and tinnitus score was lost in rats with behavioral evidence of tinnitus (Figure 3D). Tinnitus scores were normalized across different groups of tinnitus and control rats using z-scores (z-score = [actual value – Mean]/Standard deviation). These findings suggest a significant tinnitus-related loss of nAChR function which could be driven by a decreased number of nAChRs or a change in the subunit composition of the nAChRs on layer 5 PNs.

### VIP neurons receive BF cholinergic inputs and express heterogenous populations of nAChR subtypes

**Figure 4:**
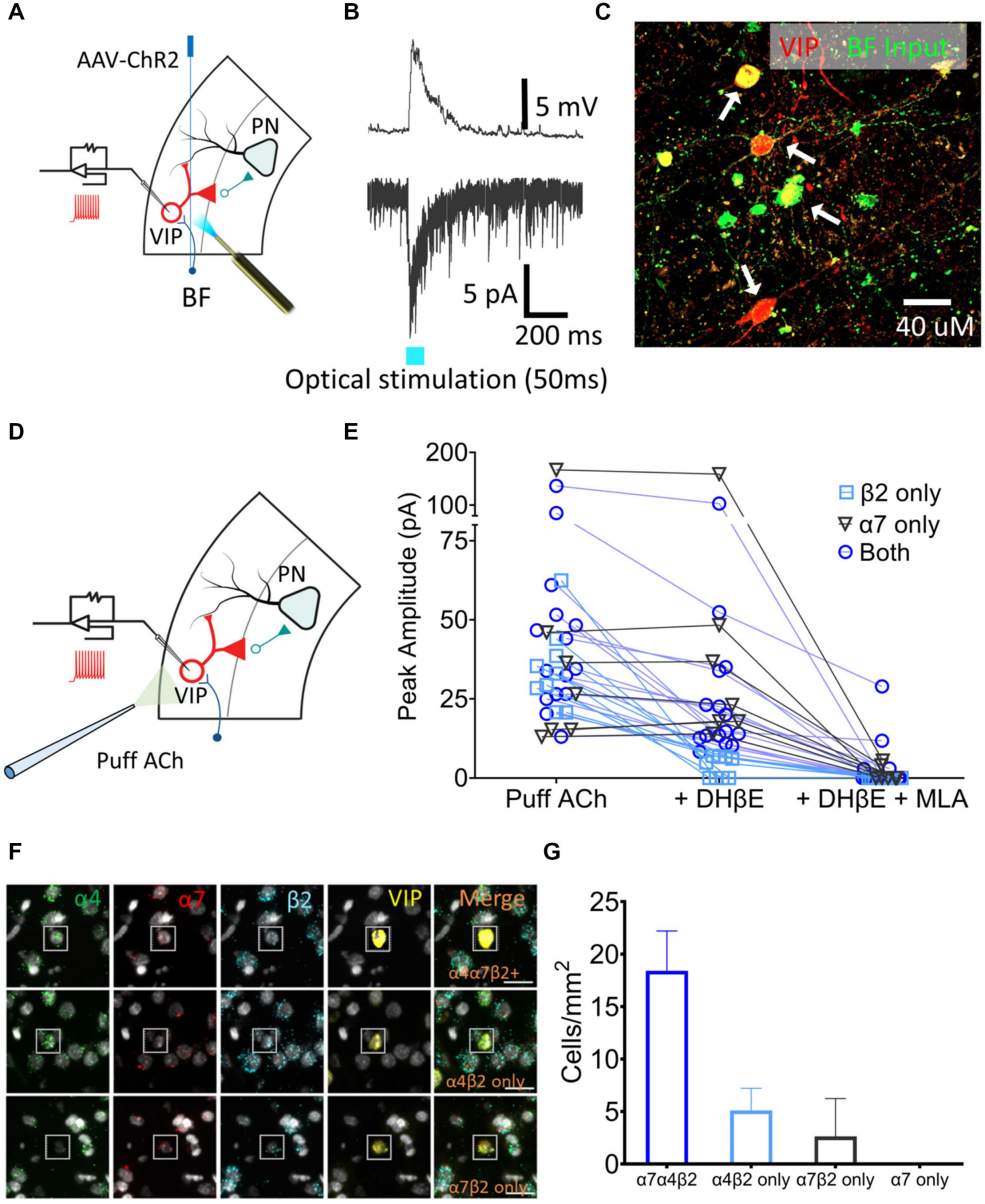
A1 VIP neurons receive cholinergic inputs from BF terminals and express a heterogenous population of nAChRs. **(A)** Illustration showing injection of an AAV-ChR2 viral vector (*AAV-CaMKIIa-hChR2(H134R)-EYFP*) into the BF of *VIP^Cre^:Rosa26^tdTomato^* LE rats, and whole-cell patch-clamp recordings from A1 VIP neurons (red) after expression of virus. **(B)** Optical stimulation BF cholinergic terminals evoked a sharp depolarizing current in current-clamped (top) and an inward current in voltage-clamped (bottom) recordings. **(C)** BF terminals (EYFP, green) contacted the soma and dendrites of A1 VIP neurons (tdTomato, red; white arrows show colabelled cells). **(D)** Illustration showing whole-cell patch-clamp recordings from VIP neurons used to examine responses to puffed ACh. **(E)** nAChR responses were categorized as expressing β2-containing heteromeric nAChRs only (n = 9 neurons, blue square), α7 homomeric nAChRs only (*n* = 7 neurons, gray triangle) or both β2-containing and α7 nAChRs (*n* = 15 neurons, blue circle) using the subunit-selective blockers (DhβE and/or MLA). **(F)** Exemplar FISH images showing nAChR transcript expression in VIP neurons (insets). Top row, co-expression of three nAChR transcripts (α4,α7, and β2); Middle row, co-expression of α4 and β2 transcripts only; Bottom row, co-expression of α7 and β2 transcripts only. **(G)** Average cell density of VIP neurons expressing specific nAChR transcript combinations in a single cell.

**Figure 5:**
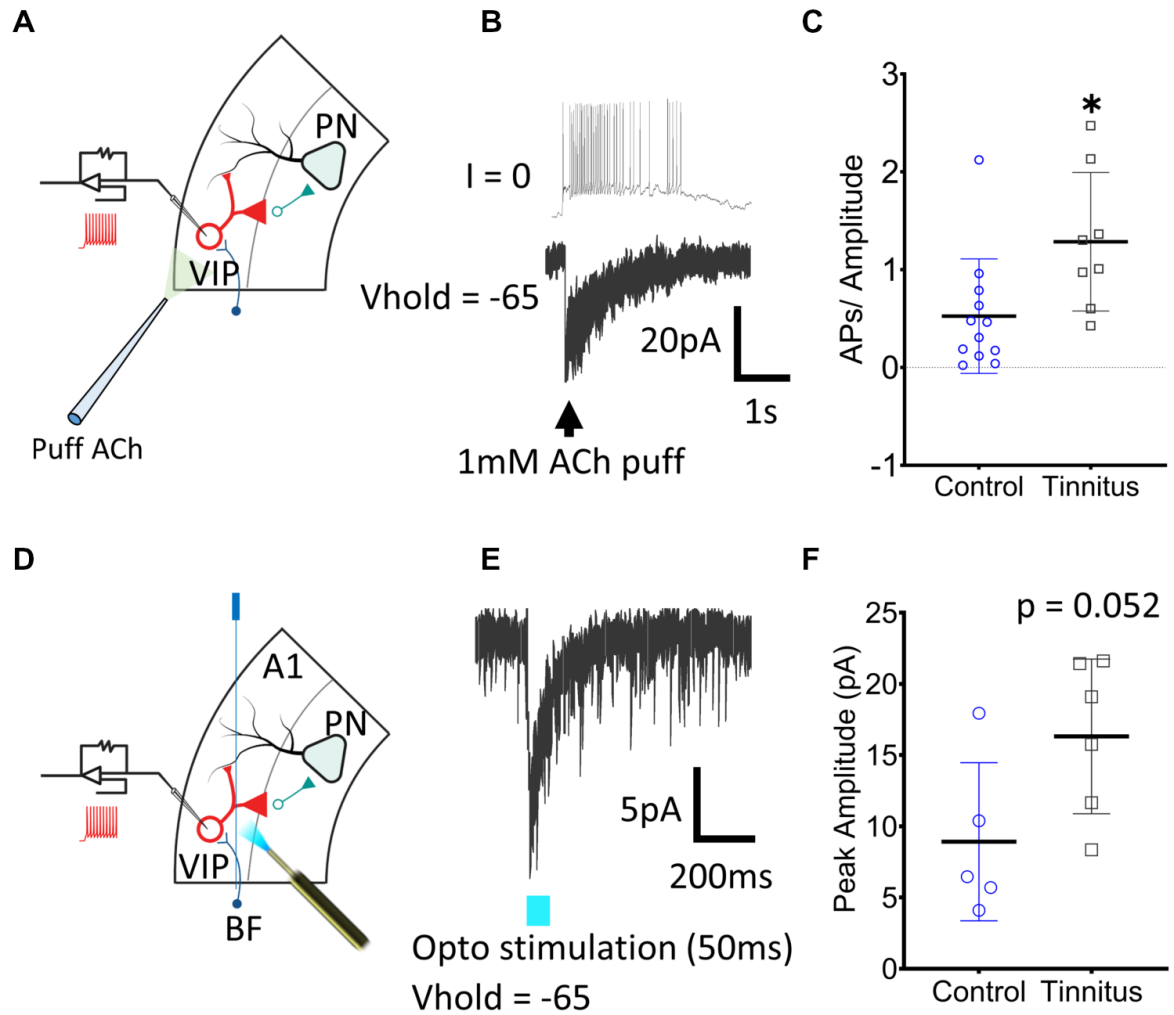
A1 VIP neurons show tinnitus-related increased nAChR excitability. **(A)** Illustration showing whole-cell patch-clamp recordings from A1 VIP neurons with puffed ACh. **(B)** Exemplar traces show a train of APs generated by puffed ACh in control animals. **(C)** A significant increase in nAChR excitability of VIP neurons was observed when APs were normalized to the amplitude of the evoked current (t (18) = 2.62, *p* = 0.017, Student’s *t*-test, control, *n* = 12 neurons, 3 rats, tinnitus, *n* = 8 neurons, 3 rats). **(D)** Whole-cell patch-clamp recordings were obtained from VIP neurons with optical stimulation of BF terminals as shown in the illustration. **(E)** Optical stimulation of BF terminals evoked a sharp inward current in voltage-clamped recordings. **(F)** A trend towards a tinnitus-related increase in BF input was observed but the difference was non-significant (t (9) = 2.2, *p* = 0.052, Student’s *t*-test, control, *n* = 5 neurons, 2 rats, tinnitus, *n* = 6 neurons, 2 rats). ^*^ *p* < 0.05.

VIP neurons are key regulators of layer 5 PN excitability. To examine the presence of cholinergic input onto the VIP neurons, *AAV-CaMKIIa-hChR2(H134R)-EYFP*, which contains a fusion protein of EYFP and channel rhodopsin (ChR2) expressed under the control of the neuronal-specific *CaMKIIa* promoter, was injected in the BF of *VIP^Cre^:Rosa26^tdTomato^* rats and the AAV was allowed to express for 3–4 weeks. Acute 300 μm slices containing A1 were sectioned, VIP neurons were identified using tdTomato expression and whole-cell patch-clamp recordings were performed (Figure 4A). nAChR-mediated depolarizing currents were seen from 11 out of 14 VIP neurons from control rats when BF A1 terminals were optically stimulated to release ACh in the presence of bath-applied atropine, DNQX, and AP5 (muscarinic, AMPA and NMDA receptor antagonists, respectively) (Figure 4B). Inputs from BF neurons expressed EYFP proximal to the soma and dendrites of VIP neurons expressed tdTomato (Figure 4C). We next measured nAChR-evoked currents using a potassium gluconate internal solution (ECl ~ −65 mV, Vhold −65 mV) with 1 mM ACh puffed 30 μm away from a VIP soma using a pneumatic picospritzer at 7 psi for 10 ms (Figure 4D). Puffed ACh evoked a sharp depolarization from VIP neurons generating a series of action potentials which can be seen in Figure 5B. ACh-evoked responses mediated by different receptor subtypes were isolated using the selective blockers dihydro-β-erythroidine (DhβE) (β2-containing nAChR heteromeric receptors) and methyllycaconitine (MLA) (α7-containing nAChR homomeric receptors) (Figure 4E). Three populations of VIP neurons were identified based on their nAChR response properties following selective blockade. One population of VIP neurons (7/31) appeared to primarily express α7 homomeric nAChRs, a second population (9/31) expressed β2-containing nAChRs, while a third population (15/31) expressed both β2-and α7-containing nAChRs (Figure 4E). The expression of nAChR subunit transcripts was examined in A1 VIP neurons using multiplexed fluorescent *in situ* hybridization (FISH) (Figures 4F,G). Most VIP neurons co-expressed α4, β2, α7 nAChR transcripts, while smaller populations of VIP neurons co-expressed only α4 and β2 transcripts or only α7 and β2 transcripts. These findings suggest that the VIP neurons showing homomeric nAChR pharmacology may be driven by either α7 homomeric nAChR-or α7β2 heteromeric nAChR-mediated responses, both of which are blocked by MLA, but not DHβE (Wu et al., 2016). Additional studies will be required to better characterize VIP neuron nAChR pharmacology and to elucidate the functional relevance of different nAChR subtypes (see discussion).

### VIP neurons show tinnitus-related increases in nAChR-evoked excitability

*In vitro* whole-cell voltage-and current-clamp recordings using potassium gluconate internal solution (ECl ~ −65 mV) were used to collect current clamp responses from VIP interneurons to 1 mM puffed ACh, 30 μm from the neuron being studied (Figure 5A). ACh evoked strong depolarization from VIP neurons generating trains or bursts of action potentials (APs) (Figure 5B). The number of APs generated in current-clamp was normalized to the evoking current in voltage-clamped recordings (Figure 5C) (*t* (18) = 2.62, *p =* 0.017, Student’s *t*-test). VIP neurons from rats with behavioral evidence of tinnitus showed a significant increase in nAChR-mediated excitability (Figure 5C). Tinnitus-related VIP neuronal changes in endogenous nAChR signaling were examined by optically stimulating BF terminals. *AAV-CaMKIIa-hChR2(H134R)-EYFP* was injected in the BF of *VIP^Cre^:Rosa26^tdTomato^* rats as described in the methods (Figures 5D,E). While not statistically significant, we observed a trend towards increased nAChR signaling with endogenously released ACh in tinnitus animals (Figure 5F) (*t* (9) = 2.2, *p =* 0.052, Student’s *t*-test). Collectively these findings suggest that VIP neurons from animals with behavioral evidence of tinnitus show increased nAChR-evoked excitability.

### Sazetidine-A and varenicline desensitize nAChRs, increasing the strength of inhibitory inputs onto layer 5 PNs in tinnitus animals

**Figure 6:**
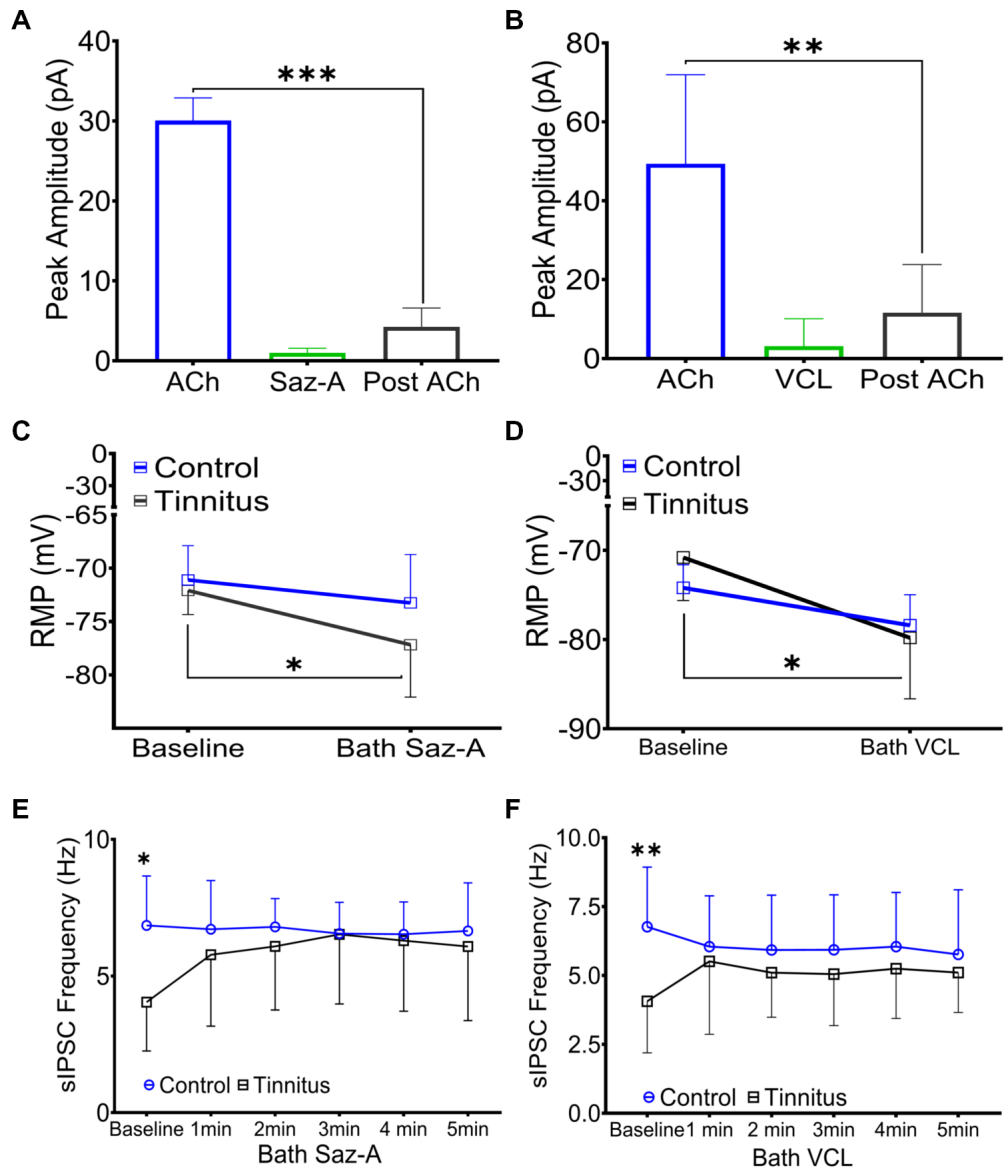
Sazetidine-A and varenicline desensitize nAChRs and increase the activity of inhibitory input neurons. **(A,B)** Whole-cell patch-clamp recordings in response to 1 mM puffed ACh, 1 μM puffed sazetidine-A or varenicline, or 1 mM puffed ACh (2 min post-sazetidine-A/varenicline). Sazetidine-A and varenicline showed no nAChR action when puffed 30 μm away from layer 5 PNs (sazetidine-A, baseline-post-sazetidine-A, *F* (2,6) = 54, *p* < 0.001, *n* = 3 neurons, 1 rat; varenicline, baseline-post-varenicline, *F* (2, 12) = 12.82, *p* = 0.0044, *n* = 5 neurons, 1 rat, one-way ANOVA with a Bonferroni post-hoc test). **(C)** Layer 5 PNs from tinnitus animals were hyperpolarized by bath application of sazetidine-A (500 nM, Control, baseline vs. bath sazetidine-A, *p =* 0.99, *n* = 9 neurons, 2 rats; Tinnitus, baseline vs. bath sazetidine-A, *p =* 0.022, *n* = 11 neurons, 2 rats; two-way ANOVA with Bonferroni post-hoc test). No measurable sazetidine-A change was observed in control animals. **(D)** Varenicline hyperpolarized layer 5 PNs in tinnitus animals (500 nM, Control, baseline vs. bath varenicline, *p =* 0.99, *n* = 5 neurons, 2 rats, tinnitus, baseline vs. bath varenicline, *p =* 0.048, *n* = 5 neurons 1 rat, two-way ANOVA with Bonferroni post-hoc test) while no measurable change was observed in control animals. **(E)** Layer 5 PNs in tinnitus animals showed a significant increase in the frequency of sIPSCs 5 min after bath-applied sazetidine-A (Control, baseline vs. bath sazetidine-A 1–5 min, *p* = ~0.9, *n* = 10 neurons, 3 rats; Tinnitus, baseline vs. bath sazetidine-A 1 min, *p* = 0.018, 2 min, *p* = 0.024, 3 min, *p* = 0.012, 4 min, *p* = 0.016, and 5 min, *p* = 0.05, *n* = 13 neurons, 4 rats, Control baseline vs. Tinnitus baseline, *p* = 0.04, two-way repeated measures ANOVA with Tukey’s multiple comparison test). **(F)** Layer 5 PNs in tinnitus animals showed a significant increase in the frequency of sIPSC gradually after bath-applied varenicline (Control, baseline vs. bath VCL 1–5 min, *p* = ~0.99, n = 13 neurons, 4 rats; Tinnitus, baseline vs. bath VCL 1 min, *p* = 0.18, 2 min, *p* = 0.04, 3 min, *p* = 0.05, 4 min, *p* = 0.01, and 5 min, *p* = 0.02, *n* = 10 neurons, 3 rats, Control baseline vs. Tinnitus baseline, *p* = 0.0028, two-way repeated measures ANOVA with Tukey’s multiple comparison test). ^*^ *p* < 0.05, ^**^ < 0.01, ^***^ < 0.001.

Sazetidine-A and varenicline have been previously described as potent nAChR partial desensitizing agonists (Coe et al., 2005; Xiao et al., 2006; Rollema et al., 2009; Rezvani et al., 2013). We examined the effects of 1 μM sazetidine-A and 1 μM varenicline puffed 30 μm from layer 5 PNs in patch-clamp recordings using potassium gluconate internal solution (ECl ~ −65 mV, Vhold −65). Puffed ACh evoked strong inward currents from A1 layer 5 PNs expressing α4β2 heteromeric nAChRs and α7 homomeric nAChRs (Figures 3A,B, 6A,B). Similar to previous reports, puffed sazetidine-A and varenicline failed to evoke postsynaptic inward currents from PNs from control or tinnitus animals (Figures 6A,B), confirming that these compounds have little agonist activity (Coe et al., 2005; Xiao et al., 2006). When 1 mM ACh was puffed onto the same patched PNs 2 min after sazetidine-A or varenicline, there was a significant decrease in the ACh-evoked current, (sazetidine-A, *F*(2, 6) = 54, *p* < 0.001; varenicline, *F* (2, 12) = 12.82, *p* = 0.044, One-way ANOVA with Bonferroni post-hoc test). These findings were similar to previous reports in other tissues suggesting significant sazetidine-A/varenicline-mediated desensitization of nAChRs (Coe et al., 2005; Xiao et al., 2006). Following the collection of 5 min of baseline spontaneous excitatory postsynaptic currents (sEPSCs) recorded from layer 5 PNs, an additional 5 min of sEPSCs were recorded following bath application of 500 nM sazetidine-A or varenicline. Sazetidine-A or varenicline application significantly hyperpolarized layer 5 PNs from tinnitus animals (sazetidine-A, *F* (1,35) = 8.46, *p =* 0.022, varenicline, *F* (1, 16) = 9.85, *p =* 0.048, two-way ANOVA with a Bonferroni post-hoc test). No significant changes in resting membrane potential was seen in control animals with similar bath application of sazetidine-A or varenicline (Figures 6C,D). This led us to hypothesize that sazetidine-A and varenicline may indirectly normalize/increase inhibitory tone/input onto PNs recorded from tinnitus animals. To examine the direct action of sazetidine-A or varenicline on inhibitory input neurons, sazetidine-A or varenicline were bath applied during whole-cell patch-clamp recordings from layer 5 PNs (CsCl internal solution; ECl ~ 0 mV, Vhold −65 mV). Changes in the frequency and amplitude of spontaneous inhibitory postsynaptic currents (sIPSCs) were monitored over a period of 10 min (5 min baseline following a 5 min bath application of 500 nM sazetidine-A or varenicline). Baseline measurements revealed significant tinnitus-related decreases in sIPSC frequency impacting layer 5 PNs (Figures 6E,F) (sazetidine-A, *F* (1, 108) = 5.35, *p =* 0.019, varenicline, *F* (1,138) = 10.55, *p* = 0.006, Two-way ANOVA with a Bonferroni post-hoc test). Bath application of sazetidine-A or varenicline resulted in a gradual increase in sIPSC frequency onto layer 5 PNs in tinnitus animals (Figures 6E,F). Control animals showed no significant changes in sIPSC frequency onto layer 5 PNs following 1 min of bath-applied sazetidine-A or varenicline (Figures 6E,F).

### Sazetidine-A and varenicline reduced psychophysical evidence of tinnitus in a dose-dependent manner

**Figure 7:**
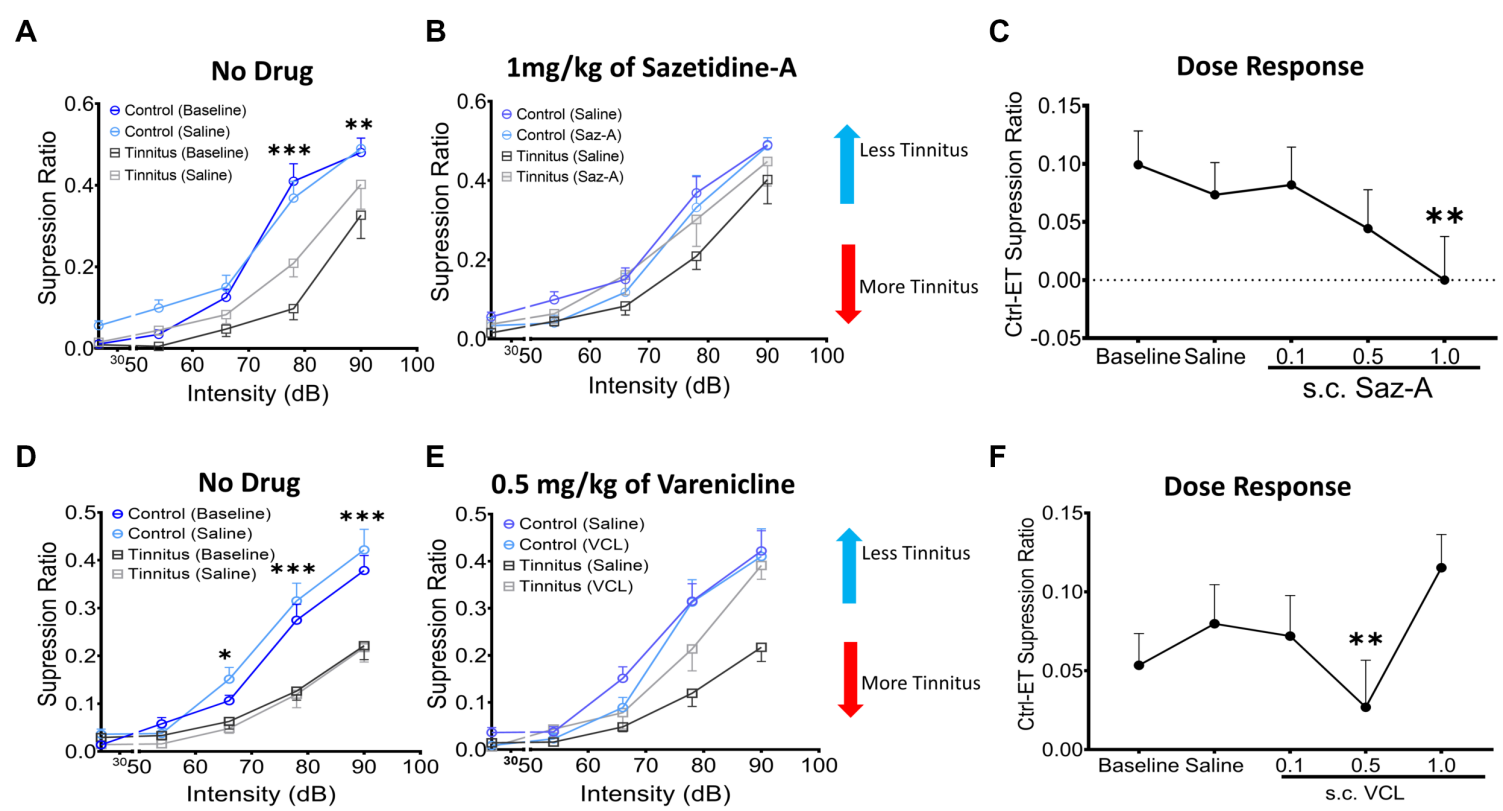
Sazetidine-A and varenicline ameliorate tinnitus-behavior. **(A,D)** Baseline suppression ratios with saline injection for control and tinnitus animals in a 20 kHz discrimination test prior to sazetidine-A or varenicline administration. At baseline, a significant separation in suppression ratios was observed between control and tinnitus animals (sazetidine-A baseline, *F* (1, 45) = 20.29, *p* < 0.0001; varenicline baseline, *F* (1,224) = 2.78, *p* < 0.008, two-way ANOVA with Bonferroni post-hoc test). **(B,E)** The suppression ratio between control and tinnitus groups became non-significant following 1 week (5 days) of daily pre-treatments (an hour before behavioral tests) with sazetidine-A (*F* (1,45) = 0.7, *p* = 0.99, *n* = 5 control, 6 tinnitus) or varenicline (*F* (1,70) = 1.17, *p* = 0.28, n = 8 control, 8 tinnitus, Two-way ANOVA). **(C,F)** Three different doses, 0.1, 0.5, 1 mg/kg of each drug were examined in these same animals and among the doses tested, 1 mg/kg of sazetidine-A and 0.5 mg/kg of varenicline were found most effective in improving animals’ tone discrimination behavior (saline vs. 1 mg/kg sazetidine-A, *p* = 0.007, saline vs. 0.5 mg/kg varenicline, *p* = 0.0014, one-way ANOVA with Bonferroni’s post-hoc test). ^*^ *p* < 0.05, ^**^ < 0.01, ^***^ < 0.001.

Sazetidine-A or varenicline were administered subcutaneously (sc.) to control and ET/tinnitus animals 1 h prior to behavioral testing. Increasing doses of sazetidine-A up to 1 mg/kg, effectively ameliorated behavioral evidence of tinnitus (Figures 7A,B). Administration of 1 mg/kg sazetidine-A resulted in the noise-exposed tinnitus group and unexposed control group no longer showing statistical separation between psychometric functions but showing partially overlapping psychometric functions (*F* (1, 45) = 0.7, *p =* 0.99, two-way ANOVA with a Bonferroni post-hoc test). Lower doses of sazetidine-A, 0.5 mg/kg and 0.1 mg/kg, reduced the difference between the tinnitus and control psychometric functions but the functions remained statistically different (Figure 7C). Figure 7C plots sazetidine-A dose against the suppression difference score (unexposed control – exposed tinnitus) and shows the reduced evidence of tinnitus at 0.5 and 1.0 mg/kg doses of sazetidine-A (1.0 mg/kg, *F* (4, 20) = 7.27, *p =* 0.007, one-way ANOVA with a Bonferroni post-hoc test). Likewise, sc. administration of 0.5 mg/kg varenicline effectively decreased behavioral evidence of tinnitus (Figures 7D,E) (*F* (4,30) = 12.43, *p =* 0.0014, one-way ANOVA with a Bonferroni post-hoc test). Neither lower nor higher doses of varenicline, 0.1 mg/kg and 1 mg/kg, statistically improved behavior evidence of tinnitus (Figure 7F).

## Discussion

The present studies find significant tinnitus-related disruption of nAChR signaling in A1 layer 5 PNs. In contrast, A1 inhibitory VIP neurons from tinnitus rats showed significant increases in nAChR-mediated excitability. Slice studies showed that nAChR partial desensitizing agonists, sazetidine-A and varenicline increased/normalized inhibitory input onto PNs in tinnitus animals. The same nAChR desensitizing agents, sazetidine-A and varenicline ameliorated behavioral evidence of tinnitus in our animal model of tinnitus.

### Behavioral model of psychophysical tinnitus in rats

A long-standing problem for tinnitus studies was the availability of reliable animal models. Jasterboff and colleagues in 1988 introduced a psychophysical paradigm that utilized sodium salicylate for tinnitus induction and a conditioned lick suppression paradigm in water-restricted animals for tinnitus assessment (Jastreboff et al., 1988). Bauer and Brozoski (2001) made a number of refinements to Jastreboff’s model, resulting in the noise-exposure model used in the present studies (Bauer and Brozoski, 2001). Brozoski’s model utilized a 1/3rd octave narrowband noise centered at 17 kHz to induce tinnitus in LE rats and subsequently trained in a conditioned suppression discrimination task to lever press for food pellets (see Methods). This behavioral paradigm had two major advantages that were critical to the present study: (1) Noise-exposure-induced tinnitus shared more pathological similarities with human tinnitus, as most human tinnitus is induced by peripheral damage caused by excessive noise-exposure and (2) Tinnitus can be assessed over long periods of time, making this model suitable for testing pharmacological agents over time.

### Tinnitus-related loss of nAChR signaling in A1

A tinnitus-related decline in attentional mechanisms has been described in multiple human and animal studies (Burton et al., 2012; Roberts et al., 2013; Brozoski et al., 2019). Similar to the visual cortex, neurons in the deeper layers of A1 are believed to utilize ACh when performing tasks including selective attention, arousal, learning, and memory (Godfrey et al., 2017; van Kerkoerle et al., 2017; Intskirveli and Metherate, 2021). A1 layer 5 PNs are the primary output neurons that shape ascending acoustic information projections to the inferior colliculus and medial geniculate body (Felix et al., 2018; Williamson and Polley, 2019; Blackwell et al., 2020; Kommajosyula et al., 2021). Layer 5 PNs express nAChRs and puffed ACh evokes strong depolarizing responses that can generate action potentials (Ghimire et al., 2020). Although a few studies have described tinnitus-related decreases in selective attention, it is unclear if auditory attention is a pathological hallmark or a correlative factor in severe tinnitus. Human studies suggest bimodal abnormalities of attentional function showing both loss of selective attention and inability to shift attention away from tinnitus phantom sounds in their heads (Hallam et al., 2004; Rossiter et al., 2006; Stevens et al., 2007). For example, Stevens et al. (2007) tested the cognitive and attentional abilities of patients with chronic tinnitus and found that patients with chronic tinnitus perform significantly poorer in complex and attention demanding tasks, compared to control groups. These finding suggest complex cell/circuit-specific changes at multiple auditory and non-auditory structures (Rauschecker et al., 2010; Shore et al., 2016; Makani et al., 2022). Tinnitus-related loss of nAChR-evoked response in the layer 5 PNs observed in this study may contribute to the observed decline in attentional resources seen by Brozoski et al. (2019) in this same animal model.

### Tinnitus-related increases in nAChR excitability of VIP neurons

VIP neurons are a key inhibitory neuronal subtype that regulate the activity of principal neurons, PNs, in A1 via disinhibitory mechanisms. Activation of VIP neurons suppresses the activity of PV and SOM neurons, resulting in decreased inhibition onto layer 5 PNs (Askew et al., 2019). A significant tinnitus-related increase in nAChR-evoked excitability in response to puffed ACh was observed in VIP neurons. Therefore, tinnitus-related increases in nAChR-mediated evoked excitability of VIP neurons has the potential to enhance the activity of layer 5 PNs. Similar increases in the excitability of VIP neurons were observed in the somatosensory cortex in a mouse model of chronic neuropathic pain (Cichon et al., 2017). Historically, chronic pain and tinnitus are believed to share similar pathological traits, as both are thought to be phantom percepts originating from partial deafferentation of peripheral sensory neurons (Møller, 2011; Vanneste et al., 2019). Therefore, therapeutic approaches decreasing nAChR function could decrease the activity of VIP neurons and have the potential to decrease the magnitude/perception of both phantom sensations.

### Sazetidine-A and varenicline may decrease tinnitus by increasing the activity of inhibitory input neurons

Sazetidine-A and varenicline effectively desensitized nAChRs on layer 5 PNs, with little evidence of agonist activity. Although the pharmacological properties of sazetidine-A and varenicline were not tested directly in the VIP neurons, we predict similar desensitizing nAChR responses based on our PN data and previous reports (Xiao et al., 2006; Rollema et al., 2009). Bath application of sazetidine-A and varenicline significantly increased the frequency of sIPSCs impinging on layer 5 PNs in tinnitus animals, while control animals showed no measurable changes in the activity of inhibitory PN inputs. Prolonged bath application of sazetidine-A and varenicline hyperpolarized layer 5 PNs, further supporting their role in increasing/normalizing inhibition onto PNs from rats with behavioral evidence of tinnitus. Although both of these drugs were effective in increasing the activity of inhibitory input neurons, bath-applied sazetidine-A produced a more pronounced effect on the activity of the inhibitory input neurons. The differential effects of sazetidine-A and varenicline may reflect their pharmacological profile because sazetidine-A is a more selective and more potent desensitizing agonist at α4β2 heteromeric nAChRs, while varenicline acts as a partial desensitizing agonist at α4β2 nAChRs and full agonist at α7 homomeric nAChRs (Papke et al., 2010). In animal models of chronic/neuropathic pain, a disorder that may share pathological traits with tinnitus, nAChR desensitizing agents effectively reduced pain severity (Cucchiaro et al., 2008; Møller, 2011; AlSharari et al., 2012). Increasing the activity of inhibitory neurons in the somatosensory cortex prevented the development of chronic pain and likewise increasing the activity of inhibitory neurons may have prevented the development of tinnitus in animal models (Cichon et al., 2017; Deng et al., 2020). Sazetidine-A and varenicline, similar to other nAChR partial agonists, were found to be effective in the management of formalin-induced chronic pain in animal models (Cucchiaro et al., 2008; AlSharari et al., 2012). As postulated, sc. injection of 1 mg/kg of sazetidine-A and 0.5 mg/kg of varenicline effectively ameliorated tinnitus behavior in our rat condition-suppression model.

In humans, a broad range of therapeutic agents including antidepressants, anticonvulsants, anxiolytics, muscle relaxants and many other categories of therapeutic agents have been tried for the management of tinnitus, yet not a single drug is approved by the FDA for the management of tinnitus. Many pharmacologic treatment strategies have shown significant off-target effects which narrows the use of these agents for tinnitus patients who present with comorbities including anxiety, depression, and so on (Langguth and Elgoyhen, 2012). Sazetidine-A and varenicline specifically target α4β2 heteromeric nAChRs and show similar pharmacodynamic desensitizing properties in rat and human tissue (Xiao et al., 2006; Rollema et al., 2009). We hypthesize a similar desensitization-induced increase of inhibitory input current onto the projection neurons of A1 in humans. Varenicline was formally a first-line treatment for smoking cessation and was considered clinically safe for human use. Compared to therapeutic approaches discussed above, varenicline and possibly sazetidine show only moderate off-target effects supporting their potential use in the clinical management of in tinnitus (Jiménez-Ruiz et al., 2009).

## Conclusion

Collectively, tinnitus-related loss of nAChR signaling in A1 layer 5 PNs and increased responses to nAChR signaling in VIP neurons may, in part, underpin pathological attentional deficits in tinnitus subjects. Sazetidine-A and varenicline normalized tinnitus-related losses of inhibitory input onto layer 5 PNs likely via desensitization of nAChRs located on VIP neurons. In behavioral studies, both sazetidine-A and varenicline decreased the psychophysical evidence of tinnitus in our animal model.

## Data availability statement

The raw data supporting the conclusions of this article will be made available by the authors, without undue reservation.

## Ethics statement

The animal study was reviewed and approved by Southern Illinois University School of Medicine Institutional Animal Care and Use Committee (IACUC: 41-021-007).

## Author contributions

Electrophysiology, Operant behavioral training or testing and associated imaging studies carried out in DC Neurobiology laboratories at SIU-SM. FISH studies by TH at Vanderbilt University. MG study design, data acquisition, data analysis, data interpretation, manuscript drafting, and editing. RC data analysis and interpretation manuscript editing. LL receptor binding, IHC, data collection and interpretation, manuscript editing. KB operant behavior, generation, training and testing tinnitus subjects. KW and TB study design, Operant behavior, generation, training and testing tinnitus subjects. TH generation, interpretation analysis of FISH data, and manuscript editing. BC study design, data interpretation, and manuscript editing. DC study design, data analysis, data interpretation, and manuscript editing and agrees to be accountable for all aspects of the work and will ensure that questions related to the accuracy or integrity of any part of the work are appropriately investigated and resolved. All authors contributed to the article and approved the submitted version.

## Funding

This work was supported by Department of Defense Award PR 180160, Office of Naval Research Award N000141612306 and National Institute of Health (NIH) Award DC000151 to DC and NIH Award DC015388 to TH.

## Conflict of interest

DC, TB, and BC hold a US patent (US17/428,164) for the use of sazetidine-A and varenicline for tinnitus therapy. BC is a consultant for Turner Scientific, LLC and conducts sponsored research for Decibel Therapeutics, Inc. and Otonomy, Inc.

The remaining authors declare that the research was conducted in the absence of any commercial or financial relationships that could be construed as a potential conflict of interest.

## Publisher’s note

All claims expressed in this article are solely those of the authors and do not necessarily represent those of their affiliated organizations, or those of the publisher, the editors and the reviewers. Any product that may be evaluated in this article, or claim that may be made by its manufacturer, is not guaranteed or endorsed by the publisher.
